# Neuromuscular Responses to Simulated Brazilian Jiu-Jitsu Fights

**DOI:** 10.2478/hukin-2014-0130

**Published:** 2014-12-30

**Authors:** Bruno Victor Corrêa da Silva, Bernardo Neme Ide, Mário Antônio de Moura Simim, Moacir Marocolo, Gustavo Ribeiro da Mota

**Affiliations:** 1 Human Performance and Sport Research Group, Postgraduate Program in Physical Education, Federal University of Triangulo Mineiro, Uberaba, Minas Gerais, Brazil.; 2 University Center of Belo Horizonte (Uni-BH), Department of Environmental, Biological and Health Sciences, Belo Horizonte, Minas Gerais, Brazil.; 3 Laboratory of Exercise Biochemistry, Biochemistry Department, Biology Institute, State University of Campinas, Campinas, Sao Paulo, Brazil.

**Keywords:** muscle power, combat sports, performance changes, aerobic and anaerobic metabolism

## Abstract

The aim of this study was to investigate the neuromuscular performance responses following successive Brazilian Jiu-Jitsu (BJJ) fights. Twenty-three BJJ athletes (age: 26.3 ± 6.3 years; body mass: 79.4 ± 9.7 kg; body height: 1.80 ± 0.1 m) undertook 3 simulated BJJ fights (10 min duration each separated by 15 min of rest). Neuromuscular performance was measured by the bench press throw (BPT) and vertical counter movement jump (VCMJ) tests, assessed before the 1st fight (Pre) and after the last one (Post). Blood lactate (LA) was measured at Pre, 1 min Post, and 15 min Post fights. Paired t-tests were employed in order to compare the BPT and VCMJ results. One-way ANOVA with Bonferroni post hoc tests were utilized to compare LA responses. The results revealed a significant (p < 0.05) increase in VCMJ performance (40.8 ± 5.5 cm Pre vs. 42.0 ± 5.8 cm Post), but no significant changes in the BPT (814 ± 167 W Pre vs. 835 ± 213 W Post) were observed. LA concentration increased significantly (p < 0.05) at Post, both in the 1st min (10.4 ± 2.7 mmol 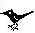
L-1) and the 15th min (6.4 ± 2.5 mmol 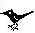
L-1) of recovery. We concluded that successive simulated BJJ fights demanded considerable anaerobic contribution of ATP supply, reinforcing the high-intensity intermittent nature of the sport. Nevertheless, no negative impact on acute neuromuscular performance (power) was observed.

## Introduction

Brazilian Jiu-Jitsu (BJJ) is a high-intensity combat sport, combining intermittent bouts of very intense anaerobic exercise, interspersed with lower-intensity periods of aerobic exercise. In BJJ, as well as in Judo, anaerobic metabolism is especially important for providing short and quick bursts of energy for attacking, defending and counterattacking, whereas the aerobic metabolism contributes to maintain high-intensity actions during the bouts providing faster recovery between them. Nowadays, BJJ techniques are also part of the training programs of mixed martial arts athletes.

The sport is also characterized by strenuous contact, demanding a high level of strength and power, combined with a well-developed aerobic, anaerobic, and buffering capacity (intra and extra muscular pH regulation) from athletes. Nevertheless, little is known about the best ways to improve sport-specific performance in BJJ during an entire season.

The consensus in literature is that the strenuous contact and high-intensity efforts may induce muscle damage, fatigue, significant acute decreases in muscle function (Warren et al., 2001), inflammatory responses (Coutts and Reaburn, 2008; Coutts et al., 2007) and increases in plasmatic activity of myofibrilar proteins such as creatine kinase and lactate dehydrogenase (Lieber et al., 2002; Warren et al., 1999). Strength, power, maximal voluntary contraction (dynamic or isometric), and the rate of force development are typically reduced immediately after exercise-induced muscle damage and fatigue, followed by linear recovery over a time-course of minutes or hours (Ide et al., 2011). Nowadays the literature emphasizes that these measures of muscle function provide the most effective methods of evaluating the magnitude of impairment, as well as the time-course of recovery, because functional impairments are immediate, prolonged and perhaps the most important symptom of muscle damage and fatigue when considering athletic performance (Ide et al., 2011).

Since neuromuscular responses after a single BJJ training session have not been thoroughly studied, the purpose of this study was to evaluate the metabolic and neuromuscular performance (power) responses to simulated BJJ bouts. Our initial hypothesis was that the high-intensity and strenuous contact nature of the sport could induce acute muscle function impairments.

## Material and Methods

### Experimental Approach to the Problem

**Figure 1:**
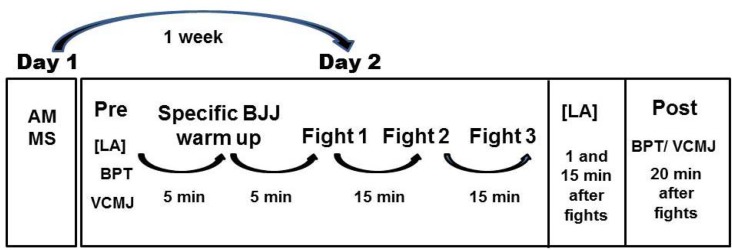
Experimental design of the study. AM = anthropometric measurements; MS = maximal strength (1RM); VCMJ = vertical counter movement jump; BPT = bench press throw; LA = blood lactate concentration

Neuromuscular performance and blood lactate responses were observed after simulated BJJ combats. Upper body power testing consisted of the bench press throw (BPT), while lower body neuromuscular performance was evaluated using the vertical countermovement jump (VCMJ). One week before the evaluations all participants were familiarized with the testing protocols and were allowed to practice the tests to minimize any neural learning effects. Anthropometric measurements and the Bench Press one repetition maximum (BP1RM) were also performed. The VCMJ and BPT were accessed at both Pre (after the warm-up) and 20 min Post BJJ fights. All participants received a standardized strong verbal encouragement during the physical tests and BJJ fights as well. Figure 1 presents the experimental design of the study.

### Participants

Twenty-three male BJJ athletes (age: 26.3 ± 6.3 years; body mass: 79.4 ± 9.7 kg; body height: 1.80 ± 0.1 m) participated in the study. The inclusion criteria included being engaged in resistance-training programs for at least one year; practicing BJJ for at least 3 years (technical level ranged from blue to black belt); training frequency of at least 4 times per week; no intake of exogenous anabolic androgenic steroids, or dietary supplements with potential effects on physical performance. Resistance-training programs typically performed by the participants aimed predominantly at strength endurance and muscle hypertrophy. All athletes were assessed during a training camp conducted in the week of the pre competitive phase and were required to refrain from strenuous exercise and consumption of alcohol, tobacco and caffeine 48 h prior and during the testing sessions. This study was approved by the Ethical Committee for Human Experiments of the Federal University of Triangulo Mineiro, Brazil (protocol 1889/2011) and was performed in accordance with the Declaration of Helsinki. In addition, all participants signed an informed consent form.

### Evaluations

#### Bench Press Maximal Strength Test

The one repetition maximum test for the Bench Press (BP1RM) exercise was employed to assess maximal strength of the participants and to prescribe the external resistance for the BPT test (30% of 1RM). The BP1RM test was conducted according to the methods previously described by Brow and Weir (2001). Before the tests the participants performed a general warm-up (3–5 min of light activity involving the muscles to be tested), followed by static stretching exercises, also for the involved musculature. Afterwards, participants performed a specific warm-up set of 8 repetitions at approximately 50% of the estimated BP1RM, followed by another set of 3 repetitions at 70% of the estimated BP1RM. Subsequent lifts were single repetitions of progressively heavier weights until the BP1RM was determined to the desired level of precision. Rest intervals of approximately 4 to 5 min were taken between each attempt to ensure phosphocreatine recovery (Glaister, 2005). The range of the number of single repetitions was 3 to 5 and the test was considered valid if the participant completed the entire lift in a controlled manner without assistance.

#### Upper Limb Power Output – the Bench Press Throw (BPT)

The BPT test employed in this study followed the procedures recommended by Comstock et al. (2011). The participants performed a specific warm-up set of 5 repetitions using only the bar (20 kg), followed by another set of 3 repetitions at 20% of the estimated 1RM. Peak power was measured using the Myotest^®^ Performance Measuring System (Myotest^®^ SA). The Myotest^®^ was clipped onto the inside portion of the Smith machine bar (Master Fitness Equipment^®^, MG, Brazil) just outside the handgrip range. The equipment was maintained in a vertical position (perpendicular to the floor) for all attempts and the peak power for each movement was obtained for the concentric portion of the movement. Three consecutive movements with 30% of BP1RM were performed. The highest value was taken for further analysis.

#### Lower Limb Power Output - Vertical Countermovement Jump (VCMJ)

The VCMJ test followed the procedures recommended by Maulder and Cronin (2005). After completing a standardized warm-up (3 non-maximal and practice trials of VCMJ, followed by lower limb stretching exercises), participants performed 3 maximal VCMJs. The participants began by standing on the designated testing leg with their toes on the starting line. They were instructed to bend their knees (approximately 120° knee angle) as quickly as possible and then jump as high as possible on both feet. Three attempts by each participant were performed, with 1 min of rest between each attempt, and the highest jump was registered for analysis. Jump height was acquired by the Myotest^®^ equipment that was allocated on the wrist of each participant, attached by a neoprene belt.

#### Simulated Brazilian Jiu-Jitsu Fights

Before participating in the fights, each athlete performed 10 min of a standardized BJJ warm-up consisting of 5 min of general stretching and 5 min of specific BJJ exercises with a work/rest ratio of 30 s each. Afterwards, each participant performed 3 BJJ fights to simulate the demands of an official competitive BJJ combat. The opponents in the fights were approximately of the same weight category and technical level. Each fight lasted 10 min with 15 min rest intervals between them. In cases where a submission occurred, the fight was continued into overtime in order to complete the initial stipulated training volume.

#### Blood Lactate Evaluations

Blood lactate responses (LA) of each participant were evaluated at Pre (before the fight and warm-up), and 1 and 15 min Post simulated fights. Lactate concentration was obtained using a portable lactate analyzer (Accusport; Boehringer Mannheim, Germany) (Bishop, 2001; Pyne et al., 2000).

### Statistical Analyses

Paired t-tests were employed to compare the power in the BPT and height of VCMJ values at Pre and Post simulated BJJ fights. One-way ANOVA with Bonferroni post hoc tests were employed to compare LA responses. The level of significance was set at 0.05. Pearson coefficient correlation was employed to analyze relationships between the tests and other variables. The correlation coefficients were interpreted using the scale of magnitudes proposed by Hopkins (www.sportsci.org): 0.1, trivial; 0.1–0.3, small; 0.3–0.5, moderate; 0.5–0.7, large; 0.7–0.9, very large; .0.9, nearly perfect. The coefficient of determination was used for interpreting the meaningfulness of the relation. All results are presented as mean and standard deviation (SD).

## Results

All athletes completed the entire BJJ simulated fights as initially proposed. The BP 1RM was 103.4 ± 22.9 kg, and thus that the intensity employed for the BPT test (30% of 1RM) was 20.7 ± 4.6 kg. The relative force for the Bench Press (BP 1RM/total body mass) was 1.3 ± 0.2 kg/kg BM.

Table 1 shows the results of correlation analysis. None of the parameters analyzed was significantly correlated to Post LA concentration (Table 1). The coefficients of determination between post LA concentration and all variables investigated were low.

### VCMJ Performance and BPT Power Output

**Figure 2:**
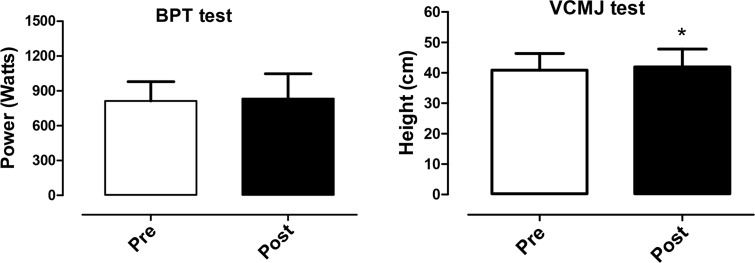
*Power output in the Bench press throwing (BPT) test and values in the Vertical counter movement jump (VCMJ) test at Pre and Post BJJ* fights. Data presented as mean and standard deviation. *Significant (p < 0.05) difference to Pre.

Significant increases (p < 0.05) of VCMJ performance (40.8 ± 5.5 cm Pre vs. 42.0 ± 5.8 cm Post) were observed. On the other hand, no significant (p < 0.05) changes for the BPT (814 ± 167 W Pre vs. 835 ± 213 W Post) test were noted. Figure 2 illustrates the changes in BPT and VCMJ tests following simulated fights.

### Blood Lactate Responses

**Figure 3:**
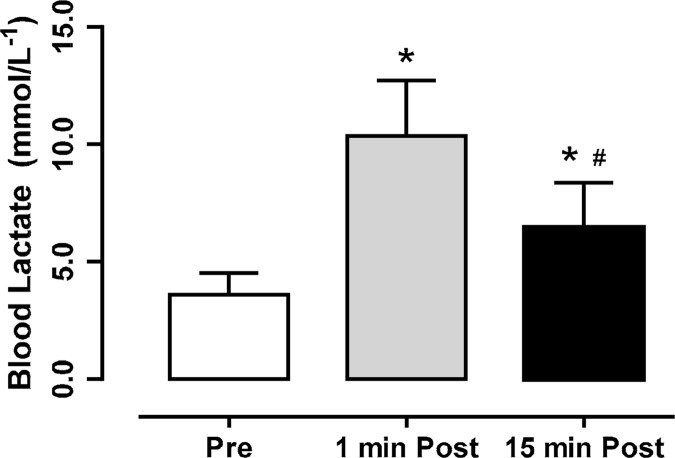
*Blood lactate concentrations at Pre, 1 and 15 min Post BJJ* fights. *Significant (p < 0.05) difference to Pre. #Significant (p < 0.05) difference to 1 min Post.

The LA increased significantly (p < 0.05), both at 1 min (10.4 ± 2.7 mmol·L^−1^) and 15 min (6.4 ± 2.5 mmol·L^−1^) Post. Figure 3 illustrates blood lactate concentration following the BJJ simulated fights.

As can be inferred from Figure 3, in each group the observed *actual* TET proves to be *significantly shorter* than the predicted TET *only after sleep* (EME_[Retest 1]_: *t* (11) = −3.901, *p*_[one-tailed]_ = .001, *d* = 1.13; MEM_[Retest 2]_: *t* (11) = −5.019, *p*_[one-tailed]_ < .001, *d* = 1.41). Also, in the EME-group this sleep-induced advantage of actual over estimated TET-performance appears to be preserved during the wake interval following sleep (EME_[Retest 2]_: *t* (11) = −1.967, *p*_[one-tailed]_ = .038; *d* = .57). In the MEM-group, however, actual and estimated TET-performance are not dissociated at all by the wake interval *preceding* sleep (MEM_[Retest 1]_: *t* (11) = −.825, *p*_[two-tailed]_ = .427, *d* = .25). According to these results, the significant reductions in TET reported for both experimental groups in the course of a 24-hr retention period cannot be attributed to merely continuing practice. Rather, and in support of our central hypothesis, they provide evidence for true sleep-related *offline-learning*, independently of sleep being administered during the first or during the second half of a 24-hrs retention interval.

## Discussion

The present study aimed to observe the neuromuscular responses following successive simulated BJJ fights. Our main findings were that the simulated BJJ fights significantly increased Post LA concentration (Figure 3); on the other hand, no performance decrements in the VCMJ and BPT tests were observed (Figure 2). Decreases in LA concentration 15 min Post were also observed, but no significant correlations between LA, neuromuscular responses (BPT and VCMJ) and body composition (BM and LBM) were noted (Table 1). Considering the present results, our initial hypothesis that high-intensity intermittent and strenuous contact nature of BJJ would induce acute performance decrements was not confirmed.

To the best of our knowledge, this is the first study to examine neuromuscular performance (VCMJ and BPT) post simulated BJJ fights. Our results are in agreement with others reported in literature derived from investigation of neuromuscular function post Judo fights. Bonitch-Dominguez et al. (2010) investigated changes in peak leg power post four judo bouts (5-min bouts separated by 15 min of passive rest) and their relationship with LA concentration. Their results showed no effect of successive bouts on peak leg power and they concluded that successive judo bouts produced high LA concentrations which had no effect on peak power.

Similar results were observed by Kraemer et al. (2001) that also investigated the performance responses to a simulated freestyle wrestling tournament. Throughout the study a battery of tests was performed at pre and immediately post each individual fight. Lower body power and upper body isometric strength were significantly reduced as the tournament progressed. Significant elevations in LA concentration were also observed after the bouts. The authors concluded that a wrestling tournament potentialized the physiological and performance decrements, although, a careful interpretation of these results must be done since the wrestlers investigated were adopting typical weight loss techniques (∼ 6% of total body mass). The study conclusions were that the combined effects of weight loss and fights may ultimately be reflected in the wrestler’s ability to maintain the physical performance throughout the tournament.

Additionally, Barbas et al. (2011) investigated the physiological and performance responses of well-trained adult wrestlers to a simulated one-day tournament following a typical weight loss regimen. Twelve competitive athletes completed five fights according to the official Olympic wrestling tournament regulations following a ∼ 6% weight loss. Among various performance markers used in the study, the Vertical Jump performance test presented a significant decline from baseline values only before the 4^th^ combat, but recovered prior to the 5^th^ combat (final combat) of the tournament. However, post-combat Vertical Jump performance did not differ from their respective pre values. Although the author suggested that a one-day wrestling tournament may induce significant physiological demands that may adversely affect their performance, especially during the later stages of the tournament, it could be observed that the Vertical Jump was less susceptible to decline during the course of a 1-day wrestling tournament than other performance markers.

However, it is important to highlight the differences in tests (isometric vs dynamic) with the aim to observe the neuromuscular responses following successive simulated fights, which can influence the results. For example, when maximal isometric handgrip tests were examined in Judo, wrestling and BJJ, a decrease in this variable was observed. Specifically in BJJ, Andreato et al. (2013) observed a decline in strength grip of approximately 11% and 16% for the right and left hands, respectively. Following a wrestling tournament (Kraemer et al., 2001) (subdivided in two days, three fights on day 1 and two fights on day 2), a significant reduction in a specific isometric strength test (bear hug) was found. On the other hand, the Vertical Jump was not significantly different from baseline values for any of the fights on day one. During a single-day simulated Greco-Roman wrestling tournament (Barbas et al., 2011), where athletes performed a total of five fights, isometric strength by bear hug was lower for fights 4 and 5, than the respective baseline values. The Vertical Jump decreased only after fight 4. Based on our results and on the aforementioned studies, we suggest that isometric strength for upper limbs may be more susceptible to decline after BJJ, wrestling and judo fights than power performance for upper and lower limbs. This may be explained by the fact that in grappling fights, lower limbs are more required for dynamic than isometric actions. These observations may help coaches and athletes to improve their training prescription emphasizing the specific strength manifestations for BJJ.

As stated previously, muscle function measurements are the best method for quantifying muscle damage after the cessation of physical activity and after resting for several hours until muscle recovery is completed (Byrne et at., 2004; Warren et al., 1999). The hypothesis raised for these observations involves higher mechanical stress imposed to sarcomeres, leading to structural breakdown of Z disks due to the loss of proteins such as desmin and α-actinin (Gibala et al., 2000). The vast majority of studies reported in literature is often well consolidated to correlate these events to muscle action variables (Lieber and Friden, 1999; Lieber et al., 2002) and particularly to the eccentric phase of the movement. Eccentric muscle actions result in greater evidence of damage and impairment of subsequent contractile activity than isometric or concentric actions (Byrne et al., 2004; Gibala et al., 2000; Gibala et al., 1995; Lieber et al., 2002). Keeping this in mind, we speculated that during a BJJ fight the athlete’s muscular performance was not compromised due to eccentric muscle contractions as it occurred in other combat sports. Meanwhile, despite this finding, we recognize that one limitation of the present study was not having a quantification of temporal behavior of plasma myofibrilar proteins activity.

Regarding LA concentrations, our results corroborate to other studies (Pereira et al., 2011) such as judo (Degoutte et al., 2003), wrestling (Karnincic et al., 2009) and BJJ (Vidal Andreato et al., 2012; Vidal Andreato et., 2013). Lactate is an unavoidable product of anaerobic glycolysis because the lactate dehydrogenase enzyme has a speed greater than any other enzyme of the glycolytic pathway, inducing a constant balance of the reaction pyruvate ↔ lactate to the formation of lactate (Robergs et al., 2004). In addition, the decrease in LA concentration 15 min post exercise corroborates with the works of Franchini and coworkers that used simulated fights in Judo (Franchini et al., 2009) and BJJ (Vidal Andreato et al., 2012). High LA concentration observed post fights not inducing performance decrements may be explained by recent findings in literature reporting that the release of lactate and H^+^ ions to the outside of the muscle cells constitutes a protective mechanism against intracellular pH decrease, instead of an event inducing muscle fatigue (Messonnier et al., 2007).

## Conclusion

In conclusion, the data of the present study suggest that BJJ-simulated fights request significant anaerobic contribution of ATP supply. Meanwhile, it seems that acute neuromuscular performance (muscle power) is not impaired by BJJ-simulated fights. Besides, there is no association between blood lactate concentrations post fights and neuromuscular performance. Future studies are necessary to investigate the neuromuscular responses along BJJ microcycles, to report the time course of muscle function throughout a training cycle, and different performance tests, such as strength endurance, could be applied.

## Figures and Tables

**Table 1:** Correlation analysis between Post LA concentration and other tests

Variables	r	p
LA Post and BPT Post	0.06	0.77
LA Post and VCMJ Post	0.12	0.58
LA Post and BM	0.36	0.09
LA Post and LBM	0.22	0.31

LA = blood lactate concentration; BPT = bench press throw; VCMJ = vertical counter movement jump; BM = body mass; LBM = lean body mass

## References

[b1-jhk-44-249] Barbas I, Fatouros IG, Douroudos II, Chatzinikolaou A, Michailidis Y, Draganidis D, Jamurtas AZ, Nikolaidis MG, Parotsidis C, Theodorou AA (2011). Physiological and performance adaptations of elite Greco-Roman wrestlers during a one-day tournament. Eur J Appl Physiol.

[b2-jhk-44-249] Bishop D (2001). Evaluation of the Accusport® lactate analyser. Int J Sports Med.

[b3-jhk-44-249] Bonitch-Dominguez J, Bonitch-Gongora J, Padial P, Feriche B (2010). Changes in peak leg power induced by successive judo bouts and their relationship to lactate production. J Sports Sci.

[b4-jhk-44-249] Brown LE, Weir JP (2001). ASEP Procedures recommendation I: accurate assessment of muscular strength and power. J Exerc Physiol Online.

[b5-jhk-44-249] Byrne C, Twist C, Eston R (2004). Neuromuscular function after exercise-induced muscle damage: theoretical and applied implications. Sports Med.

[b6-jhk-44-249] Casartelli N, Muller R, Maffiuletti NA (2010). Validity and reliability of the Myotest accelerometric system for the assessment of vertical jump height. J Strength Cond Res.

[b7-jhk-44-249] Comstock BA, Solomon-Hill G, Flanagan SD, Earp JE, Luk HY, Dobbins KA, Dunn-Lewis C, Fragala MS, Ho JY, Hatfield DL, Vingren JL, Denegar CR, Volek JS, Kupchak BR, Maresh CM, Kraemer WJ (2011). Validity of the Myotest(R) in measuring force and power production in the squat and bench press. J Strength Cond Res.

[b8-jhk-44-249] Coutts A, Reaburn P, Piva TJ, Murphy A (2007). Changes in selected biochemical, muscular strength, power, and endurance measures during deliberate overreaching and tapering in rugby league players. Int J Sports Med.

[b9-jhk-44-249] Coutts AJ, Reaburn P (2008). Monitoring changes in rugby league players’ perceived stress and recovery during intensified training. Percept Mot Skills.

[b10-jhk-44-249] Degoutte F, Jouanel P, Filaire E (2003). Energy demands during a judo match and recovery. British Journal of Sports Medicine.

[b11-jhk-44-249] Franchini E, de Moraes Bertuzzi RC, Takito MY, Kiss M (2009). Effects of recovery type after a judo match on blood lactate and performance in specific and non-specific judo task. Euro J Appl Physiol.

[b12-jhk-44-249] Gibala MJ, Interisano SA, Tarnopolsky MA, Roy BD, MacDonald JR, Yarasheski KE, MacDougall JD (2000). Myofibrillar disruption following acute concentric and eccentric resistance exercise in strength-trained men. Can J Physiol Pharmacol.

[b13-jhk-44-249] Gibala MJ, MacDougall JD, Tarnopolsky MA, Stauber WT, Elorriaga A (1995). Changes in human skeletal muscle ultrastructure and force production after acute resistance exercise. J Appl Physiol.

[b14-jhk-44-249] Glaister M (2005). Multiple sprint work: physiological responses, mechanisms of fatigue and the influence of aerobic fitness. Sports Med.

[b15-jhk-44-249] Ide BN, Leme TC, Lopes CR, Moreira A, Dechechi CJ, Sarraipa MF, da Mota GR, Brenzikofer R, Macedo DV (2011). Time course of strength and power recovery after resistance training with different movement velocities. J Strength Cond Res.

[b16-jhk-44-249] Karnincic K, Tocilj Z, Uljevic O, Erceg M (2009). Lactate profile during Greco-Roman wrestling match. J Sports Sci Med.

[b17-jhk-44-249] Kraemer WJ, Fry AC, Rubin MR, Triplett-McBride T, Gordon SE, Koziris LP, Lynch JM, Volek JS, Meuffels DE, Newton RU, Fleck SJ (2001). Physiological and performance responses to tournament wrestling. Med Sci Sports Exerc.

[b18-jhk-44-249] Lieber RL, Friden J (1999). Mechanisms of muscle injury after eccentric contraction. J Sci Med Sport.

[b19-jhk-44-249] Lieber RL, Shah S, Friden J (2002). Cytoskeletal disruption after eccentric contraction-induced muscle injury. Clin Orthop Relat Res.

[b20-jhk-44-249] Maulder P, Cronin J (2005). Horizontal and vertical jump assessment: reliability, symmetry, discriminative and predictive ability. Physical therapy in Sport.

[b21-jhk-44-249] Messonnier L, Kristensen M, Juel C, Denis C (2007). Importance of pH regulation and lactate/H+ transport capacity for work production during supramaximal exercise in humans. J Appl Physiol.

[b22-jhk-44-249] Pereira RF, Lopes CR, Dechechi CJ, Victor BC, Ide BN, Navarro AC (2011). Kinetics of lactate removal in Brazilian Jiu-Jitsu athletes. Rev Bras Prescr Fisiol Exerc.

[b23-jhk-44-249] Pyne DB, Boston T, Martin DT, Logan A (2000). Evaluation of the Lactate Pro blood lactate analyser. Eur J Appl Physiol.

[b24-jhk-44-249] Robergs AR, Ghiasvand F, Parker D (2004). Biochemistry of exercise-induced metabolic acidosis. Am J Physiol Regul Integr Comp Physiol.

[b25-jhk-44-249] Vidal Andreato L, Moraes SMF, Del Conti Esteves JV, Pereira RRA, Gomes TLM, Vidal Andreato T, Franchini E (2012). Physiological Responses and Rate of Perceived Exertion in Brazilian Jiu-Jitsu Athletes. Kineziologija.

[b26-jhk-44-249] Vidal Andreato L, Franchini E, Franzói de Moraes SM, Pastório JJ, da Silva DF, Esteves JV, Branco BH, Romero PV, Machado FA (2013). Physiological and technical-tactical analysis in Brazilian Jiu-jitsu competition. Asian J Sports Med.

[b27-jhk-44-249] Warren GL, Ingalls CP, Lowe DA, Armstrong RB (2001). Excitation-contraction uncoupling: major role in contraction-induced muscle injury. Exerc Sport Sci Rev.

[b28-jhk-44-249] Warren GL, Lowe DA, Armstrong RB (1999). Measurement tools used in the study of eccentric contraction-induced injury. Sports Med.

